# Relation of retinal blood flow and retinal oxygen extraction during stimulation with diffuse luminance flicker

**DOI:** 10.1038/srep18291

**Published:** 2015-12-17

**Authors:** Stefan Palkovits, Michael Lasta, Reinhard Told, Doreen Schmidl, René Werkmeister, Alina Popa Cherecheanu, Gerhard Garhöfer, Leopold Schmetterer

**Affiliations:** 1 Department of Clinical Pharmacology Medical University of Vienna Waehringer Guertel 18-20, 1090 Vienna, Austria; 2 Center for Medical Physics and Biomedical Engineering Medical University of Vienna Waehringer Guertel 18-20, 1090 Vienna, Austria; 3 Department of Ophthalmology, Emergency University Hospital 169, Splaiul Independentei St., District 5, Bucharest, Romania

## Abstract

Cerebral and retinal blood flow are dependent on local neuronal activity. Several studies quantified the increase in cerebral blood flow and oxygen consumption during activity. In the present study we investigated the relation between changes in retinal blood flow and oxygen extraction during stimulation with diffuse luminance flicker and the influence of breathing gas mixtures with different fractions of O_2_ (FiO_2_; 100% 15% and 12%). Twenty-four healthy subjects were included. Retinal blood flow was studied by combining measurement of vessel diameters using the Dynamic Vessel Analyser with measurements of blood velocity using laser Doppler velocimetry. Oxygen saturation was measured using spectroscopic reflectometry and oxygen extraction was calculated. Flicker stimulation increased retinal blood flow (57.7 ± 17.8%) and oxygen extraction (34.6 ± 24.1%; p < 0.001 each). During 100% oxygen breathing the response of retinal blood flow and oxygen extraction was increased (p < 0.01 each). By contrast, breathing gas mixtures with 12% and 15% FiO_2_ did not alter flicker–induced retinal haemodynamic changes. The present study indicates that at a comparable increase in blood flow the increase in oxygen extraction in the retina is larger than in the brain. During systemic hyperoxia the blood flow and oxygen extraction responses to neural stimulation are augmented. The underlying mechanism is unknown.

Functional hyperaemia in the brain was described in a landmark paper more than 100 years ago^1^. It refers to increased blood flow during neural stimulation to fulfil the metabolic demands of the tissue^2^. This hyperaemic response, also known as neurovascular coupling, exists in the retina as well^3^,^4^, but is a lot less studied. In healthy humans the increase in retinal blood flow during stimulation with pure luminance flicker appears to be as large as 50–60%^5^,^6^. A variety of studies have shown that flicker-induced changes in retinal and optic nerve head hemodynamics are reduced in ocular disease such as glaucoma^7^,^8^,^9^ or diabetic retinopathy^10^,^11^,^12^,^13^.

In the human brain, studies were published quantifying the relative magnitudes of stimulus-induced changes in blood flow, oxygen consumption and ATP^14^. Cerebral blood flow increased by the order of 60%, whereas oxygen consumption only changed by 15%. This is in good agreement with data in the rat showing that only approximately 1/3 of the cerebral blood flow response is required to support the increase in oxygen demand^15^. In addition, pharmacologically blocking the largest part of the hyperaemic response has little impact on the increase in oxygen consumption indicating that the pronounced vasodilatation is not required to maintain energy supply. In the human retina it has been shown that stimulation with diffuse luminance flicker increases oxygen saturation in retinal veins, but leaves oxygen saturation in retinal arteries constant^16^. Without concomitant quantitative measurements of retinal blood flow, it is, however, difficult to estimate the effect on the absolute amount of oxygen taken out of the retinal circulation, the oxygen extraction. In the present paper we refer to oxygen extraction instead of oxygen consumption of the retina, because there are two sources of oxygen in the retina, the retinal vessels and the choroidal vessels. Hence, retinal oxygen extraction refers to the oxygen consumed by retinal tissue delivered through the retinal circulation. To the best of our knowledge no previous study has characterized the effects of retinal stimulation with diffuse luminance flicker on retinal oxygen extraction in humans.

There is a long-standing discussion on whether neurovascular coupling is dependent on tissue oxygen levels. When oxygen is delivered directly to tissue, thereby increasing tissue pO_2_ levels, a modulatory role was observed and the hyperaemic response decreased with increasing oxygen levels^17^. When oxygen is, however, delivered via inhalation the rat retina reacts with pronounced vasoconstriction and the response to flicker light stimulation is fully preserved^17^. We have recently investigated the effect of diffuse luminance flicker on blood flow under FiO_2_ of 100% and found an augmented blood flow increase during 100% oxygen breathing due to a so far unknown mechanism^18^.

In the present study we investigated the response of retinal blood flow and retinal oxygen extraction to diffuse luminance flicker in healthy subjects. In addition, we investigated whether these responses are modified by inhaling gases with different FiO_2_ inducing either systemic hyperoxia or hypoxia.

## Materials and Methods

### Subjects

The study was performed in adherence to the Good Clinical Practice guidelines and to the Declaration of Helsinki including current revisions. Approval of the study protocol by the Ethics Committee of the Medical University of Vienna was obtained and all participating subjects provided written informed consent. Twenty-four healthy male (n = 12) and female (n = 12) non-smoking subjects aged 25.9 ± 3.7 years were included in this double-masked randomized three-way cross over study. A screening examination was scheduled for all participating subjects in the four weeks before the study day consisting of a physical examination including medical history, a blood draw to assess the haematological status and urinalysis. In addition, a full ophthalmological examination was performed. Subjects were excluded if any ophthalmological or general disease was diagnosed, in case of ametropia of more than 3 dioptres or if they used any medication or food supplements.

### Description of the study day

**Figure 1:**
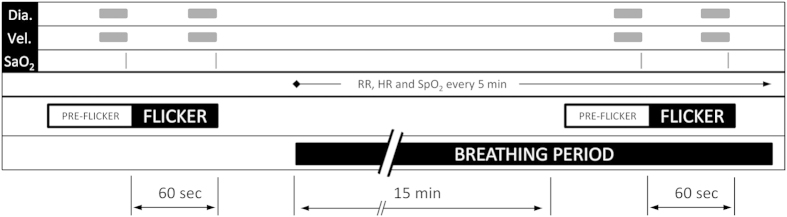
Study schedule; the same schedule was used for each gas mixture with a resting period of 120 minutes between the breathing periods.

One study day was scheduled for each subject. A study schedule is provided in Fig. 1. Topical tropicamid (Agepha®, Vienna, Austria) was administered for pupil dilatation into the study eye. After a resting period of at least 20 minutes, to ensure stabile hemodynamic conditions, baseline measurements of retinal arterial and venous diameters, retinal blood velocity and retinal arterial as well as venous oxygen saturation were performed and the retinal flicker response was determined. Oxygen partial pressure was measured using capillary blood drawn from the arterialized ear lobe. Thereafter, a sequence of three breathing periods was scheduled, each consisting of a 30 minutes period of inhalation of gas mixtures containing FiO_2_ of 12%, 15% and 100%, respectively (Messer Group GmbH, Vienna, Austria). The sequence of these breathing periods was randomized and double-masked. During the last 15 minutes of each breathing period measurements were repeated. After each breathing period a resting period of 120 minutes was scheduled. During the breathing periods systolic and diastolic blood pressure as well as heart rate and peripheral oxygen saturation were measured at 5 minutes intervals.

### Measurement of hemodynamic parameters

Systolic, diastolic and mean arterial blood pressure (SBP, DBP, MAP) were measured on the upper arm using an automated oscillometric device (Infinity Delta, Dräger, Vienna, Austria). The same device was used to continuously measure pulse rate and systemic oxygen saturation by finger pulse oximetry.

### Measurement of blood gases

Arterialized capillary blood from the ear lobe was collected from a lancet incision into a thin glass capillary tube after topical administration of nicotinate plus nonylvanillamid ointment (Finalgon®, Boehringer Ingelheim Pharma GmbH & Co. KG, Ingelheim am Rhein, Germany). Arterial pH, PCO_2_, and PO_2_ were determined using an automatic blood gas analysis system (ABL 800 Flex; Drott Medizintechnik GmbH, Wiener Neustadt, Austria).

### Measurement of retinal vessel diameter and retinal oxygen saturation

Retinal vessel diameters were measured using the Dynamic Vessel Analyser (DVA, IMEDOS Systems UG, Jena, Germany) described previously. It is a commercially available system which comprises a fundus camera (Zeiss FF 450, Jena, Germany), a video camera, a high resolution video recorder, a real time monitor and a personal computer with a vessel diameter analyzing software. The DVA allows the precise determination of retinal vessels’ diameter with a time resolution of 25 readings/s. Retinal irradiance was approximately 220 μW · cm^−2^, which is approximately 50 times lower than the maximum level allowed for constant illumination of the retina. The system provides excellent reproducibility and sensitivity (Garhöfer *et al*. 2010). In the present study, one major temporal artery and one major temporal vein were selected for measurement (D_art_, D_vein_). Assessments of retinal vessel diameters were taken between 1 and 2 disc diameters from the margin of the optic disc. The retinal flicker response was measured by recording retinal vessel diameters for 60 seconds before stimulation. Thereafter, retinal vessel diameters were measured within the last 20 seconds of a 60 second stimulation period with diffuse luminance flicker at a frequency of 12.5 Hz. The same device was used to measure retinal arterial and venous oxygen saturation based on a 2-wavelength technique before and within the final 20 seconds of flicker light stimulation (610 nm and 548 nm)^19^. The flash light intensity was set within to the recommended range (Vesselmap 1, Imedos, Jena, Gemany).

### Laser-Doppler Velocimetry

For measurement of mean retinal venous blood velocity (vel) a fundus camera based bi-directional laser Doppler system (LDV-5000, Oculix Inc., Arbaz, Switzerland) was used as described in detail previously^20^. Measurements were performed at identical locations as diameter and oxygen saturation measurements and stimulation with diffuse luminance flicker was applied in the same way as with the DVA. Like in vessel diameter and oxygen saturation analysis values from the final 20 seconds were considered for further evaluation. Analysis of data was done as outlined previously^21^,^22^ taking only those parts of the measurement for which the theoretical angle dependence was fulfilled. Blood flow in the retinal vein (Q) under study was calculated as follows:


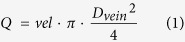


### Calculation of oxygen extraction

Oxygen content (cO_2_) in the retinal arteries and veins was estimated using Henry’s law:


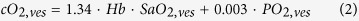


In this equation Hb is the haemoglobin concentration, and SaO_2_ is the retinal oxygen saturation in arteries and veins as measured with the DVA. Arterial PO_2_ was measured photometrically from the blood sample (Sysmex XE 500, Kobe, Japan) and venous PO_2_ from the oxygen-binding curve at a PCO_2_ level of 37 mmHg and a temperature of 37 degrees.

The retinal oxygen extraction was calculated as:


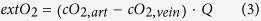


### Statistical analysis

All statistical analyses were done using the Statistica® software package (Release 6.0, StatSoft Inc., Tulsa, OK, USA). Vessel diameters and blood flow velocities before flicker (BF) were calculated as an average of the last 20 seconds before start of diffuse luminance flicker. Vessel diameters and blood flow velocities during flicker (FL) stimulation were calculated as an average of the last 20 seconds of diffuse luminance flicker. Retinal oxygen saturation was assessed prior to flicker light stimulation (BF) and within the final 20 seconds of flicker light stimulation (FL). At each point in time one fundus image was considered for further evaluation.

Flicker-induced changes in retinal outcome variables (retinal flicker response) were expressed as percentage change over baseline values ((FL − BF) × 100/BF). Shapiro–Wilk test was used to assure normal distribution of the data. A repeated measures ANOVA model was used to determine statistical significance between baseline and the breathing periods. Post hoc analysis was done using planned comparisons. All results are presented as means ± standard deviation. A p < 0.05 was considered the level of significance.

## Results

### Breathing of gas mixtures

**Figure 2:**
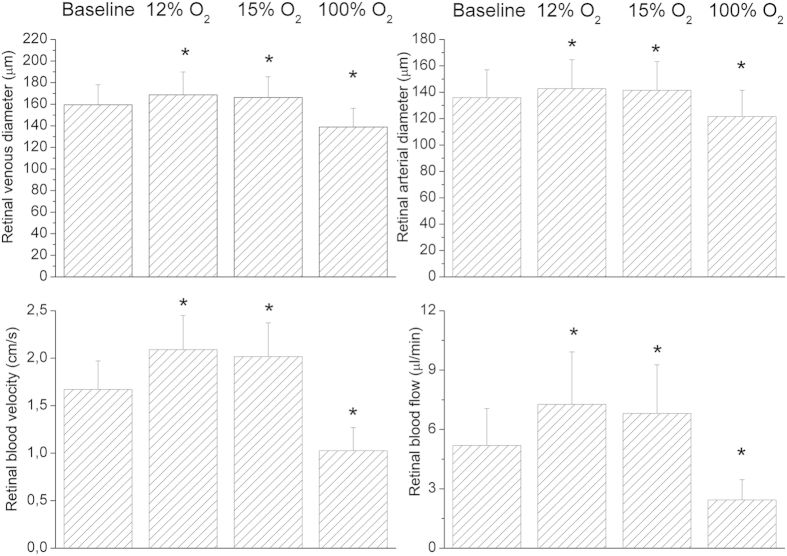
Retinal vessel diameters, retinal blood velocity and retinal blood flow at baseline and during inhalation gas mixtures with FiO_2_ of 12% 15% and 100%. Data are presented as mean and standard deviation (n = 24). Asterisks mark significant differences versus baseline.

**Figure 3:**
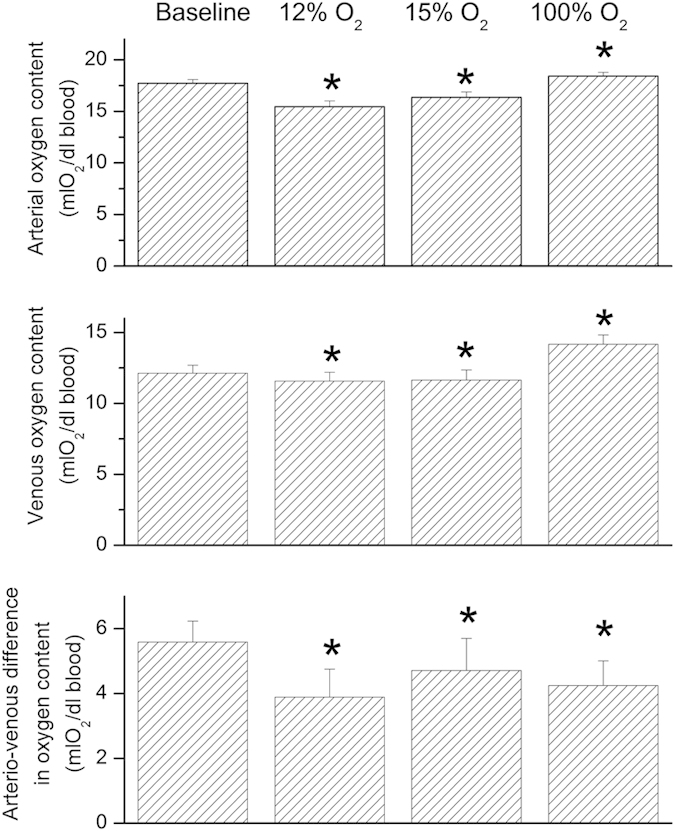
Arterial and venous oxygen content and arterio-venous difference in oxygen content at baseline and during inhalation of gas mixtures with FiO_2_ of 12% 15% and 100%. Data are presented as mean and standard deviation (n = 24). Asterisks mark significant differences versus baseline.

**Figure 4:**
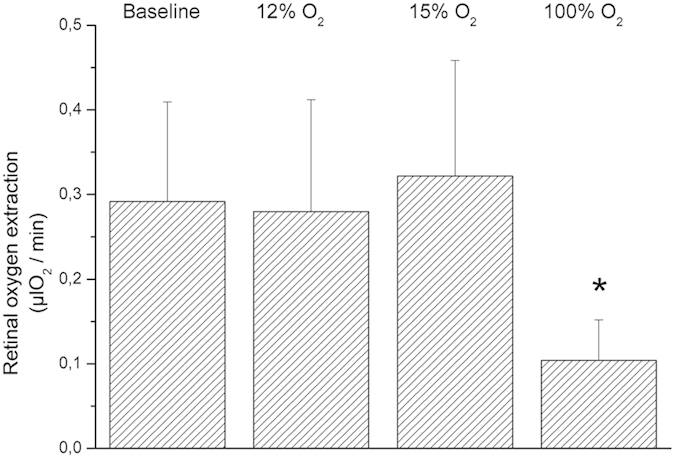
Retinal oxygen extraction at baseline and during inhalation of gas mixtures with FiO_2_ of 12% 15% and 100%. Data are presented as mean and standard deviation (n = 24). Asterisks mark significant differences versus baseline.

Systemic hemodynamic parameters as obtained during the study are shown in Table 1. None of the gas mixtures induced any significant effect on systemic hemodynamic parameters, except for peripheral oxygen saturation (p < 0.001). Breathing gas mixtures with reduced FiO_2_ induced vasodilation in retinal arteries and veins (Fig. 2). This effect was significant versus baseline (p < 0.001) and more pronounced to 12% FiO_2_ as compared with 15% FiO_2_ (p = 0.002). By contrast, D_art_ (p < 0.001) and D_vein_ (p < 0.001) decreased during 100% oxygen breathing. During systemic hypoxia vel (p < 0.001) as well as flow (p < 0.001) increased. Again, the effect was more pronounced during 12% oxygen breathing than during 15% oxygen breathing (vel: p = 0.002, flow: p < 0.001). Figure 3 shows the effects of different breathing conditions on oxygen content. cO_2,art_ was reduced versus baseline during both 12% and 15% oxygen breathing (p < 0.001). This effect was more pronounced at the lower FiO_2_ (12%, p < 0.001). Both, 12% (p < 0.001) and 15% (p < 0.001) oxygen breathing also reduced cO_2vein,_ to a comparable degree (p = 0.38). During 100% oxygen breathing cO_2art_ increased by 1.7 ± 0.4% and cO_2vein_ increased by 3.8 ± 1.1% (p < 0.001). All gas mixtures reduced cO_2diff_ versus baseline (p < 0.001). This effect was comparable at FiO_2_ of 12% and 100% and less pronounced at 15% (p < 0.001). Retinal oxygen extraction was comparable between baseline, 12% and 15% oxygen extraction (p = 0.33), but was strongly reduced during 100% oxygen breathing (p < 0.001, Fig. 4).

### Effects of diffuse luminance flicker

**Figure 5:**
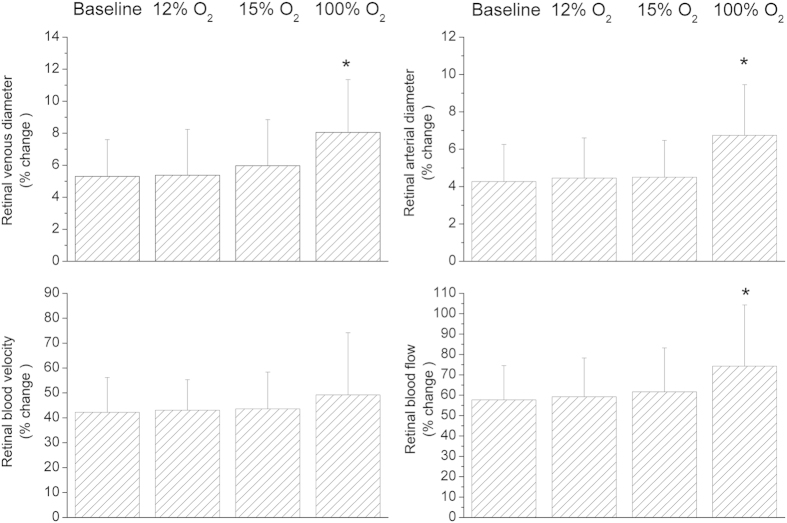
Flicker-induced changes in retinal vessel diameters, retinal blood velocity and retinal blood flow at baseline and during inhalation of gas mixtures with FiO_2_ of 12%, 15% and 100%. Data are presented as mean and standard deviation (n = 24). Asterisks mark significant differences versus baseline.

The effect of diffuse luminance flicker on retinal hemodynamic parameters is presented in Fig. 5. During all conditions stimulation with diffuse luminance flicker caused retinal arterial and venous vasodilation (p < 0.001 each). This effect was comparable between baseline conditions, 12% and 15% oxygen breathing, but was augmented during 100% oxygen breathing (p = 0.003). Retinal blood velocity showed a pronounced flicker induced increase, which was comparable between all breathing conditions (p = 0.12). Retinal blood flow increased during diffuse luminance flicker by approximately 55% during baseline, 12% oxygen breathing and 15% oxygen breathing (p = 0.67). The increase in retinal blood flow in response to diffuse luminance flicker was more pronounced during systemic hyperoxia (p = 0.017).

**Figure 6:**
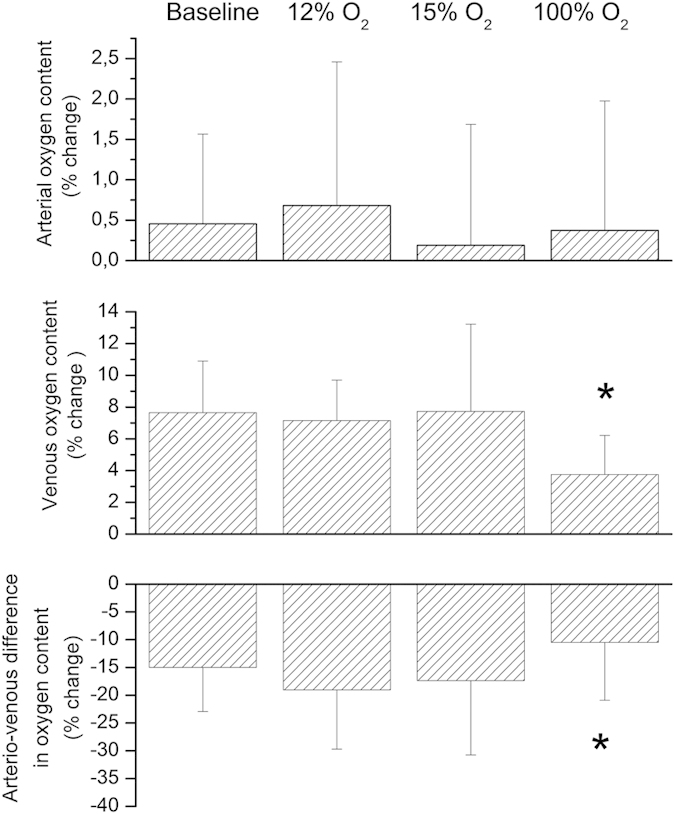
Flicker-induced changes in arterial and venous oxygen content and arterio-venous difference in oxygen content at baseline and during inhalation of gas mixtures with FiO_2_ of 12% 15% and 100%. Data presented as mean and standard deviation (n = 24). Asterisks mark significant differences versus baseline.

**Figure 7:**
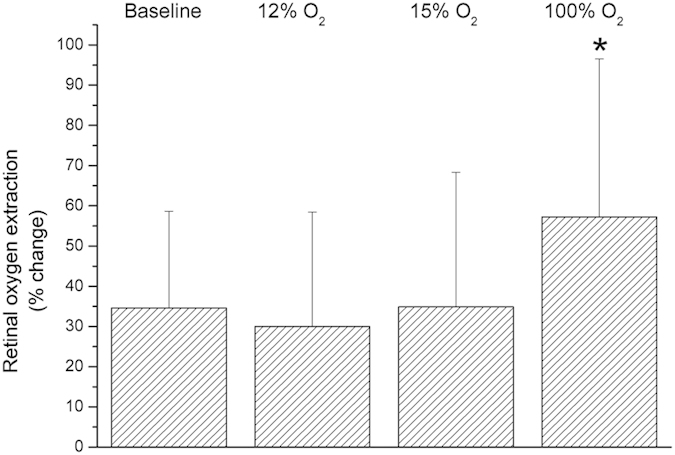
Flicker-induced changes in retinal oxygen extraction at baseline and during inhalation of gas mixtures with FiO_2_ of 12% 15% and 100%. Data are presented as mean and standard deviation (n = 24). Asterisks mark significant differences versus baseline.

**Figure 8:**
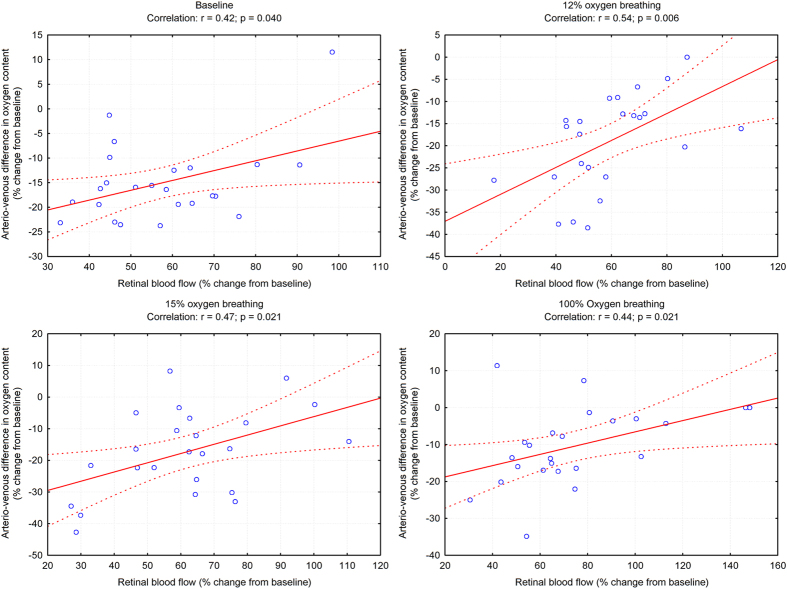
Correlation between flicker-induced changes in retinal blood flow and arterio-venous difference in oxygen content at baseline and during inhalation of gas mixtures with FiO_2_ of 12% 15% and 100%. Data are presented as mean and standard deviation (n = 24). Asterisks mark significant differences versus baseline.

Figure 6 illustrates effects of flicker light stimulation on oxygen content variables during each breathing period. Diffuse luminance flicker did not change cO_2art_ during any of the flicker stimulation periods (p = 0.34). Venous oxygen content increased during flicker stimulation (p < 0.001), an effect that was less pronounced during inhalation of 100% oxygen (p = 0.23). Hence, cO_2diff_ decreased in response to diffuse luminance flicker during all breathing conditions (p < 0.001), but less so during systemic hyperoxia (p = 0.009). Retinal oxygen extraction during flicker light exposure increased by approximately 35% during baseline conditions, 12% and 15% oxygen breathing (p < 0.001, Fig. 7). The increase in retinal oxygen extraction was significantly larger during 100% oxygen breathing as compared to breathing room air (p = 0.002). As shown in Fig. 8 the flicker-induced increase in retinal blood flow was positively correlated with the change in cO_2diff_ during all breathing conditions.

## Discussion

To the best of our knowledge this is the first study in humans to quantify retinal oxygen extraction in response to stimulation with diffuse luminance flicker. Our data indicate that an increase in retinal blood flow of approximately 55% is associated with an increase in retinal oxygen extraction of approximately 35%. In addition, our data indicate that during systemic hyperoxia as induced by 100% oxygen breathing the flicker response of retinal blood flow and retinal oxygen extraction is augmented.

In the brain the relation between cerebral blood flow, oxygen consumption, and lactate production in the human visual cortex was studied using MR technology. The increase in cerebral blood flow was comparable to the data in the present study (52–65%), but the increase in oxygen consumption (12–17%) was much smaller than in the retina^14^,^15^. In addition, the percent change in cerebral blood flow was negatively associated with the percent change in oxygen consumption, which is in contrast to our correlation analysis presented in Fig. 8. One previous study quantified retinal oxygen consumption during stimulation with diffuse luminance flicker in the rabbit`s retina by cannulating an artery and a vortex vein^23^. Using this technique an increase of 15% was seen in response to diffuse luminance flicker. Comparison with the present study is difficult, because the rabbit retina is entirely nourished by the choroidal circulation, blood flow was not quantified and the stimulus was applied at 4 Hz, which may induce less vasodilation than the 12.5 Hz employed in the present study^24^,^25^.

In the brain it was thought that the large increase in blood flow induced by activity is required to ensure the relatively small increase in oxygen consumption based on mathematical modeling^26^. When, the large increase in activity-induced cerebral blood flow is partially blocked pharmacologically, the increase in oxygen consumption is unaffected arguing against this hypothesis^15^. Whether this also holds true for the retina is unknown. Several studies reported that flicker-induced vasodilatation can be blocked^27^,^28^,^29^ but none of these studies quantified oxygen consumption or extraction. In the brain it has been hypothesized that the large increase in blood flow during activity is required to maintain blood flow during conditions of greater energy need that can occur pathologically^2^. Whether our results indicate that this reserve is lower in the retina than in the brain remains to be proven. Recent data in the rat retina indicate that flicker stimulation evoked more pronounced dilations in the intermediate layer capillaries than in the superficial and deep layer^30^. This suggests that most of the increase in retinal oxygen consumption occurs in the neuronal somata and synapses of the inner retina.

The present study indicates that during systemic hypoxia inner retinal oxygen tension is well regulated due to the increase in retinal blood flow. This is in keeping with several previous studies in animals and humans^21^,^31^,^32^,^33^. During this condition neither the hyperaemic response to diffuse luminance flicker nor the response in oxygen extraction is altered. By contrast, breathing 100% oxygen augmented the flicker-induced increase in retinal blood flow and retinal oxygen extraction in the present study. The former result is in keeping with our recent observation in healthy subjects^18^, but was not observed in rat experiments^17^.

During systemic hyperoxia the retinal hemodynamic response is complex. In keeping with a large variety of previous studies 100% oxygen breathing causes a pronounced decrease in retinal blood flow^34^,^35^,^36^. This is associated with a reduction in arterio-venous oxygen saturation and a decrease in retinal oxygen extraction that has again been reported previously^37^. The most likely explanation for this result is that during systemic hyperoxia excess oxygen is diffusing from the choroid towards the inner retina. This hypothesis is supported by the absence of a choroidal blood flow response to 100% oxygen breathing^38^,^39^ as well as the form of oxygen profiles in different retinal depths using microelectrodes^40^,^41^,^42^.

The mechanism underlying increased hyperaemic response to stimulation with diffuse luminance flicker during 100% oxygen breathing is unknown. One potential explanation is that retinal vessels are constricted and hence the activity dependent vasodilator stimulus starts at a different vascular tone. Alternatively the very low retinal oxygen extraction itself may contribute. During 100% oxygen breathing inner retinal oxygen gradients will change potentially also affecting oxygen transport during neuronal activity. Finally, it may well be that the activity of enzymes that are involved in the production of mediators of the hyperaemic response such as arachidonic acid metabolites may be dependent on the level of oxygen^17^,^29^. Little is, however, known about the mechanisms mediating activity-induced vasodilation in the human retina.

The present study has several limitations that need to be considered. The 2-wavelength spectroscopic approach for measuring oxygen saturation in retinal vessels critically depends on the calibration process. For the present device this was done by comparison with retinal vessel reflectance spectra with a 2 nm resolution^19^. Any mistake in this calibration process will lead to errors in absolute SO_2_ levels, but will not affect changes induced by flicker-stimulation of breathing of gas mixtures with different FiO_2_. The present study estimated local retinal arterial pO_2_ and Hb from systemic measurements and venous pO_2_ from previously published curves, but the error introduced by these assumptions is less than 1%^37^. Finally the values for oxygen extraction as presented in this report cannot be considered absolute, because it is unknown whether the blood supplied by a specific artery is fully drained by the adjacent vein in the human retina. This does, however, not affect the conclusion on relative changes during hypoxia, hyperoxia or flicker stimulation.

In conclusion, the hyperaemic response seems to be required to fulfil the oxygen demand in the human retina. In addition, our results indicate that increasing systemic pO_2_ by breathing pure oxygen alters the hyperaemic response of retinal vessels to stimulation with flicker light. Although the exact reason for this altered flicker response is unclear, our data support the hypothesis that neuro-vascular coupling in the retina is modulated by oxygen.

## Additional Information

**How to cite this article**: Palkovits, S. *et al*. Relation of retinal blood flow and retinal oxygen extraction during stimulation with diffuse luminance flicker. *Sci. Rep*. **5**, 18291; doi: 10.1038/srep18291 (2015).

## Figures and Tables

**Table 1:** Systemic hemodynamic parameters during breathing of the different gas mixtures (n = 24) SBP – systolic blood pressure; DBP – diastolic blood pressure; MAP – mean arterial pressure; PR – pulse rate; SpO_2_ – peripheral oxygen saturation (pulse oximetric module).

	Baseline	12% O_2_	15% O_2_	100% O_2_	p-value
SBP (mmHg)	119 ± 8	120 ± 9	120 ± 8	119 ± 10	P = 0.40
DBP (mmHg)	68 ± 8	67 ± 8	69 ± 8	68 ± 8	P = 0.20
MAP (mmHg)	85 ± 8	84 ± 8	86 ± 8	85 ± 7	P = 0.11
PR (beats per minute)	63 ± 7	63 ± 6	63 ± 7	63 ± 8	P = 0.79
SpO_2_ (%)	98.5 ± 1	98 ± 2	98 ± 2	99.8 ± 0.1	P < 0.001
